# Toll-like receptor 7 deficiency protects apolipoprotein E-deficient mice from diet-induced atherosclerosis

**DOI:** 10.1038/s41598-017-00977-0

**Published:** 2017-04-12

**Authors:** Cong-Lin Liu, Marcela M. Santos, Cleverson Fernandes, Mengyang Liao, Karine Iamarene, Jin-Ying Zhang, Galina K. Sukhova, Guo-Ping Shi

**Affiliations:** 1 Department of Cardiology, Institute of Clinical Medicine, the First Affiliated Hospital of Zhengzhou University, Zhengzhou, China; 2 Department of Medicine, Brigham and Women’s Hospital and Harvard Medical School, Boston, MA 02115 USA

## Abstract

Toll-like receptor 7 (TLR7) mediates autoantigen and viral RNA-induced cytokine production. Increased TLR7 expression in human atherosclerotic lesions suggests its involvement in atherogenesis. Here we demonstrated TLR7 expression in macrophages, smooth muscle cells (SMCs), and endothelial cells from mouse atherosclerotic lesions. To test a direct participation of TLR7 in atherosclerosis, we crossbred TLR7-deficient (*Tlr7* ^−/−^) mice with apolipoprotein E-deficient (*Apoe* ^−/−^) mice and produced *Apoe* ^−/−^ *Tlr7* ^−/−^ and *Apoe* ^−/−^ *Tlr7* ^+/+^ littermates, followed by feeding them an atherogenic diet to produce atherosclerosis. Compared to *Apoe* ^−/−^ *Tlr7* ^+/+^ mice, *Apoe* ^−/−^ *Tlr7* ^−/−^ mice showed reduced aortic arch and sinus lesion areas. Reduced atherosclerosis in *Apoe* ^−/−^ *Tlr7* ^−/−^ mice did not affect lesion macrophage-positive area and CD4^+^ T-cell number per lesion area, but reduced lesion expression of inflammatory markers major histocompatibility complex-class II and IL6, lesion matrix-degrading proteases cathepsin S and matrix metalloproteinase-9, and systemic serum amyloid A levels. TLR7 deficiency also reduced aortic arch SMC loss and lesion intima and media cell apoptosis. However, TLR7 deficiency did not affect aortic wall elastin fragmentation and collagen contents, or plasma lipoproteins. Therefore, TLR7 contributes to atherogenesis in *Apoe* ^−/−^ mice by regulating lesion and systemic inflammation. A TLR7 antagonist may mitigate atherosclerosis.

## Introduction

Toll-like receptors (TLRs) are pattern recognition receptors that recognize distinct evolutionarily conserved structures on pathogens, termed pathogen-associated molecular patterns (PAMPs) and play essential roles in innate immunity^1, 2^. Recent studies suggest an involvement of TLRs in atherosclerosis^3–5^. However, the role of TLRs in atherosclerosis has been controversial. TLR3 and TLR4 activation promoted atherosclerosis^6^. Deficiency of TLR2, TLR3, and TLR4 protected low-density lipoprotein receptor-deficient (*Ldlr* ^−/−^) mice^6–9^ and apolipoprotein E-deficient (*Apoe* ^−/−^) mice^10^ from atherosclerosis. However, these TLRs have also been suggested to play protective roles in murine atherosclerosis. TLR4 signaling protected *Apoe* ^−/−^ mice from periodontal pathogen-induced atherosclerosis^11^. In *Apoe* ^−/−^ mice, TLR3 deficiency accelerated the onset of atherosclerosis^12^. Absence of TLR9 also protected *Apoe* ^−/−^ mice from spontaneous atherosclerosis^13^.

While TLR2 and TLR4 are localized on cell surface, TLR3, TLR7, and TLR9 are localized to the endolysosomal membrane where these TLRs mediate autoantigen and bacterial and viral nucleic acid infection-induced inflammatory cytokine production. For example, viral single-strand RNA (ssRNA) binding on TLR7^14^ is required for B cell proliferation, and such activity of TLR7 is specific for RNA-containing antigens^15^. TLR stimulation by microbial infection results in elevated production of IL6 and other cytokines that block regulatory T-cell *de novo* generation^16^ and function^17^, which play protective roles in atherosclerosis^18, 19^. The role of TLR7 in atherosclerosis has also been controversial. In normal human arteries, including aorta, carotid, iliac, mesenteric, subclavian, and temporal, there was negligible expression of TLR7^20^. However, human atherosclerotic lesions contained elevated levels of TLR7^21^, suggesting an involvement of TLR7 in atherosclerosis. TLR7 signaling is mediated by MyD88, which activates downstream interferon regulatory factor 5 (IRF5) and IRF7 for the production of inflammatory cytokines (e.g. IL6 and TNF-α) and type I interferon (e.g. IFN-α), respectively^22^. In a femoral artery cuff placement-induced neointima thickness model in hypercholesterolemic ApoE*3-Leiden mice, TLR7 expression was increased 14 days after the surgery. Blocking of TLR7 signaling with its antagonist reduced neointimal formation, luminal stenosis, and foam cell formation by greater than 60% with concurrent reduction of arterial wall macrophage infiltration. Stimulation of macrophages with the TLR7 ligand imiquimod enhanced TNF-α expression^23^. Similar observations were obtained in collar placement-induced carotid artery atherosclerosis-like lesion development in hypercholesterolemic rabbits. TLR7 activation with imiquimod increased carotid artery lesion intima area, lipid deposition, and lesion macrophage and lymphocyte contents^24^. However, primary cultured human aortic wall endothelial cells (ECs) and smooth muscle cells (SMCs) produced chemokines IL8 and E-selectin in responding to ligands of TLR3 (Poly I:C), TLR4 (lipopolysaccharide), and TLR5 (flagellin), but do not respond to TLR7 ligand ssRNA^25^. In *Apoe* ^−/−^ mice fed a chow diet, TLR7 deficiency increased aortic root atherosclerotic lesion area and lesion macrophage accumulation, with reduced lesion SMC and collagen contents. In the peripheral blood, TLR7 deficiency led to increased plasma monocyte chemoattractant protein-1 (MCP-1) and increased inducible nitric oxide synthase (iNOS)-positive M1 macrophage contents. Macrophages from these mice also produced more MCP-1, TNF-α, IL6, and IL10 after stimulation with a TLR2 ligand lipoteichoic acid^21^. In lupus-prone *Fasl* ^*gld/gld*^ *Apoe* ^−/−^ mice, deficiency of IRF5 increased aortic root and arch lesion area and plasma levels of triglyceride, cholesterol, phospholipids, and non-esterified fatty acids, with concurrent reduction of plasma IL10 levels. Bone marrow macrophages from IRF5-deficient mice also showed reduced IL10 production in response to TLR7 ligands R848 or R837^26^. Therefore, the exact role of TLR7 in atherosclerosis remains uncertain and may depend on different experimental model systems.

In this study, we used TLR7-deficient (*Tlr7* ^−/−^) mice and atherogenic diet-fed *Apoe* ^−/−^ mice to test whether TLR7 deficiency affects atherogenesis differently from *Apoe* ^−/−^ mice not on an atherogenic diet or mice on a *Fasl* ^*gld/gld*^ lupus background.

## Materials and Methods

### Mice and experimental model

Both *Apoe* ^−/−^ mice (C57BL/6, N = 15, #002052) and *Tlr7* ^−/−^ mice (C57BL/6, N > 10, #008380) were purchased from the Jackson Laboratory (Bar Harbor, ME, USA) and crossbred to generate *Apoe* ^−/−^ *Tlr7* ^−/−^ mice and *Apoe* ^−/−^ *Tlr7* ^+/+^ littermates. Eight to ten-week-old male mice were used to induce atherosclerosis by feeding mice a high cholesterol (1.25%) atherogenic diet (#D12108c, Research Diets Inc. New Brunswick, NJ). After 3 months on this diet, mice from both groups were sacrificed with carbon dioxide narcosis, followed by cardiac puncture blood collection, heart and aortic arch tissue harvest. Aortic root was prepared by cutting away about 70% of the ventricles, and embedding the up portion of the heart that contained the aortic root in optimum cutting temperature (OCT) for sectioning as described previously^27^. Six µm aortic root sections were prepared for oil-red O (ORO) staining to determine atherosclerotic lesion sizes. To analyze atherosclerotic lesion in the aortic arch, we used longitudinal sections from the whole arch embedded in OCT. We collected only sections that contained all three branches (brachiocephalic artery, left common carotid artery, and left subclavian artery) as we reported^28^. Aortic arch sections were also stained with ORO to determine atherosclerotic lesion sizes. We can typically cut 8–10 serial numbered slides (3 sections per slide) and select for each staining the slides with the same lesion location, which corresponds to the same slide serial number. For example, we used one serial numbered slide for Mac-3, CD4, and major histocompatibility complex class II (MHC-II) for all mice, and used another same serial numbered slides from all mice for ORO staining. This approach gives central location of each lesion in each aortic arch with the maximal comparability of quantitative data. All animal procedures conformed to the Guideline for the Care and Use of Laboratory Animals published by the US National Institutes of Health and were approved by the Harvard Medical School Standing Committee on Animals (protocol # 03759).

### Immunohistological analysis

Serial aortic arch cryostat cross-sections were used for immunostaining to detect TLR7 (1:150, #ab45371, Abcam, Cambridge, MA), macrophages (Mac-3, 1:900, #553322, BD Biosciences, San Jose, CA), CD4^+^ T cells (CD4, 1:90, #553043, BD Biosciences), MHC-II (1:250, #556999, BD Biosciences), elastin (Modified Verhoeff Van Gieson Elastic Stain Kit, #HT25A-IKT, Sigma-Aldrich, St. Louis, MO), collagen (0.1% Sirius Red; #09400, Polysciences Inc., Warrington, PA), SMC (α-actin, 1:750, #F3777, Sigma-Aldrich), IL6 along with rabbit IgG isotype (IL6, 1:50, #ab6672, Abcam), IL4 (IL4, 1:50, #ab11524, Abcam), TGF-β (1:300, #sc-146, Santa Cruz Biotechnologies, Inc, Dallas, TX), TLR2 (1:50, #ab1655, Abcam, Cambridge, MA), cathepsin S (CatS) (1:100)^29^, and matrix metalloproteinase-9 (MMP-9) (1:400, #AB804, Chemicon International, Temecula, CA). Apoptotic cells in lesions were determined with the *in situ* apoptosis detection kit according to the manufacturer’s instructions (#S7100, Millipore, Billerica, MA). Immunostained slides were visualized with either the liquid diaminobenzidine (DAB) substrate chromogen system (#K3468, DAKO, Carpinteria, CA) and counter-stained with methyl green (#H-3402, Vector Laboratories, Inc, Burlingame, CA) (TGF-β, IL-4, IL-6, and TLR7), or with 3-amino-9-ethyl carbazole (#K3464, AEC) (DAKO) and counter-stained with Gill’s hematoxylin solution (GHS316, Sigma-Aldrich) (macrophage, MHC-II, CD4^+^ T cell and SMC). Elastin degradation, collagen content, and media SMC accumulation were graded according to the grading keys that were described previously^30, 31^. CD4^+^ T cells and TUNEL-positive apoptotic cells were counted manually in a blinded fashion and presented as numbers per aortic section. Images of the relative macrophage, MHC-II, IL6, IL4, and TGF-β contents within the aortas were captured by a Microscope VS120 Whole Slide Scanner (Olympus) and quantified by measuring the immunostaining signal-positive areas using computer-assisted image analysis software (Image-Pro Plus; Media Cybernetics, Bethesda, MD). All mouse experiments were performed, and data were analyzed in a blinded fashion by at least two observers.

### Immunofluorescent double staining

Cell type (macrophage, SMC, and EC)-specific expression of TLR7 in mouse atherosclerotic lesions was determined by immunofluorescent double staining using rabbit anti-mouse TLR7 monoclonal antibody (1:100, ab45371, Abcam) together with rat anti-mouse Mac-2 monoclonal antibody (macrophages, 1:100, CL8942AP, CEDARLANE, Burlington, NC), purified rat anti-mouse CD31 monoclonal antibody (EC, 1:900, #553370, BD Biosciences), and FITC-conjugated mouse anti-α-smooth muscle actin monoclonal antibody (SMC, 1:500, F3777, Sigma-Aldrich). Mouse aortic arch lesion macrophage and SMC apoptosis was also detected by immunofluorescent double staining with Alexa Fluor® 594-conjugate cleaved caspase-3 (Asp175) (D3E9) rabbit monoclonal antibody (1:200, #8172, Cell Signaling Technology, Danvers, MA) together with rat anti-mouse Mac-2 monoclonal antibody or FITC-conjugated mouse anti-α-smooth muscle actin monoclonal antibody as mentioned above. Images were collected under an Olympus FluoView™ FV1000 Confocal Microscope.

### Plasma protein measurements

Mouse plasma was collected after mice were fasted overnight and stored at −80 °C until analysis. Plasma IL6 (#88-7064-88), IFN-γ (#88-7314-88) (eBioscience, Inc., San Diego, CA), and serum amyloid A (SAA) (#MSAA00, R&D Systems, Minneapolis, MN) levels were determined using ELISA kits according to the manufacturers’ instructions. Plasma total cholesterol, triglyceride, and high-density lipoprotein (HDL) levels were determined using reagents from Pointe Scientific (Canton, MI) and the level of low-density lipoproteins (LDL) was calculated as the following formula: LDL = total cholesterol − HDL − (triglycerides/5) as previously reported^32^. Investigators were blinded to the sources of samples during the assays.

### Statistical analysis

All mouse data were expressed as mean ± SEM. Due to our small sample sizes and often skewed data distributions, we performed a pairwise non-parametric Mann-Whitney test followed by Bonferroni corrections to examine the statistical significances. SPSS 16.0 was used for analysis.

## Results

### TLR7 expression in mouse atherosclerotic lesions

**Figure 1:**
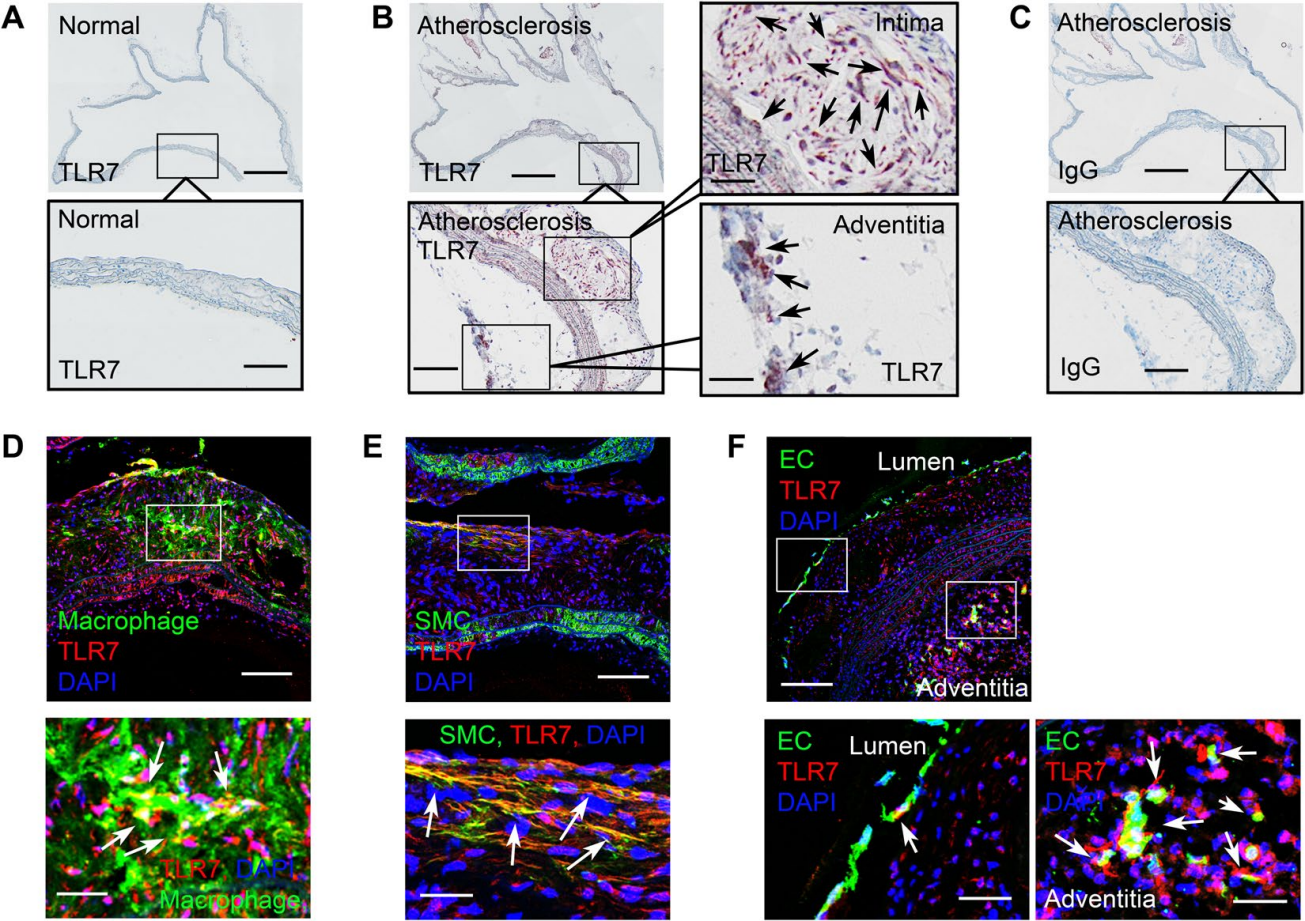
TLR7 expression in mouse atherosclerotic lesions. (**A)** Rabbit-anti-mouse TLR7 monoclonal antibody immunostaining of a normal mouse aortic arch. Scale: 1 mm, inset scale: 200 µm. (**B**) Rabbit-anti-mouse TLR7 monoclonal antibody immunostaining of an aortic arch from an *Apoe* ^*−/−*^ mouse with atherosclerosis showed TLR7 expression in intima and adventitia. Scale: 1 mm, inset scales: 200 and 68 µm. Arrows indicate TLR7-positive cells. (**C**) Rabbit IgG isotype immunostaining of a parallel aortic arch from an *Apoe* ^−/−^ mouse with atherosclerosis. Scale: 1 mm, inset scale: 200 µm. (**D–F**) Immunofluorescent double staining of mouse aortic arch atherosclerotic lesions with antibodies against TLR7 and cell type markers (Mac-2, α-actin, and CD31) for macrophage, SMC, and EC from both the lumen and adventitia microvessels. Scale: 100 µm, inset scale: 25 µm. Arrows in the insets indicate TLR7-positive macrophages (**D**), SMCs (**E**), and ECs in lumen and adventitia (**F**).

Prior studies revealed elevated TLR7 expression in human atherosclerotic lesions^21^, but negligible TLR7 in normal human arteries^20^. We obtained similar observations in mouse atherosclerotic lesions. TLR7 immunostaining did not detect TLR7 expression in normal mouse aorta (Fig. 1A). In contrast, rabbit anti-mouse TLR7 antibody revealed elevated TLR7 expression in the intima and adventitia in aortic arches from *Apoe* ^−/−^ mice that had consumed an atherogenic diet for 3 months (Fig. 1B). Immunostaining of a parallel section using a rabbit IgG isotype, as negative control, revealed negligible signal (Fig. 1C).

Immunofluorescent double staining of mouse aortic arch atherosclerotic lesions from *Apoe* ^−/−^ mice demonstrated TLR7 expression in macrophages (Fig. 1D, left two panels), SMCs (Fig. 1E, middle two panels), and ECs in both the lumen and adventitia microvessels (Fig. 1F, right three panels).

### TLR7 deficiency reduced atherosclerosis and lesion inflammation

**Figure 2:**
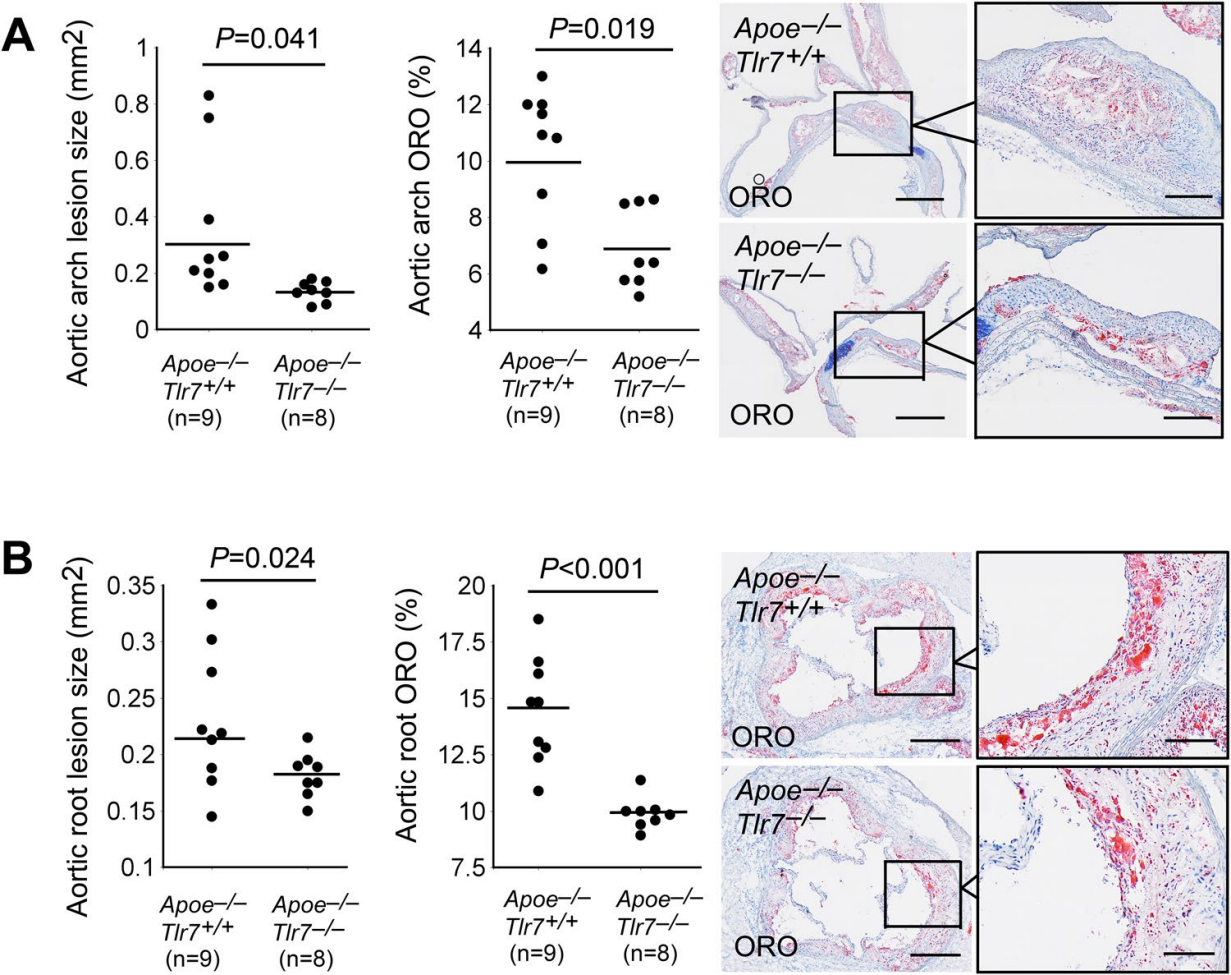
TLR7 deficiency reduces atherosclerotic lesion size and lesion lipid deposition in male *Apoe* ^−/−^ mice after 3 months of an atherogenic diet. (**A)** Aortic arch lesion size (left), ORO staining detected lipid deposition (middle), and aortic arch ORO staining representative images. Scale: 1 mm, inset scale: 200 µm. (**B**) Aortic root lesion size (left), ORO staining detected lipid deposition (middle), and aortic root ORO staining representative images. Scale: 1 mm, inset scale: 200 µm. Numbers (in parentheses) and genotypes of mice are indicated.

To test a direct role of TLR7 in atherosclerosis, we generated *Apoe* ^−/−^ *Tlr7* ^−/−^ and *Apoe* ^−/−^ *Tlr7* ^+/+^ littermate control mice. To induce atherosclerosis, we fed 8 to 10 weeks old male mice an atherogenic diet for 3 months. Mouse bodyweights or blood pressures did not differ between the two types of mice before or after the atherogenic diet. We assessed atherosclerotic lesion sizes in both the aortic arch and aortic root by staining frozen sections with ORO. In aortic arches, ORO staining revealed significantly smaller atherosclerotic lesions and lipid deposition areas from *Apoe* ^−/−^ *Tlr7* ^−/−^ mice than those from *Apoe* ^−/−^ *Tlr7* ^+/+^ control mice (Fig. 2A). We obtained similar conclusion when mouse aortic roots were analyzed. ORO staining also demonstrated significantly smaller atherosclerotic lesions and lipid deposition areas in aortic roots from *Apoe* ^−/−^ *Tlr7* ^−/−^ mice than those from *Apoe* ^−/−^ *Tlr7* ^+/+^ control mice (Fig. 2B).

**Figure 3:**
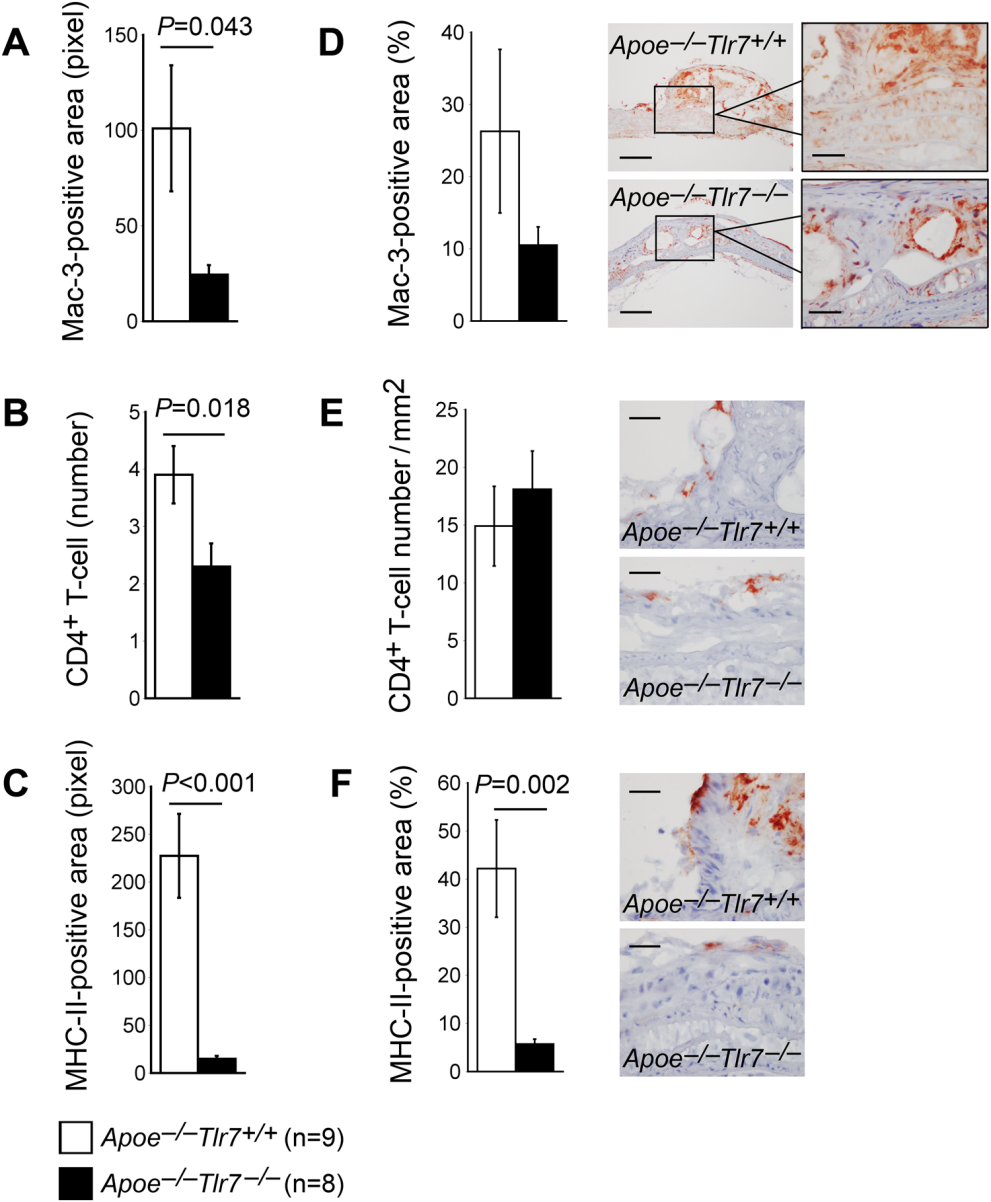
TLR7 deficiency and aortic arch atherosclerotic lesion inflammatory cell accumulation in male *Apoe* ^−/−^ mice. (**A)** Mac-3-positive area in pixel. (**B)** Lesion CD4^+^ T-cell number. (**C**) Lesion MHC class-II-positive area in pixel. (**D**) Mac-3-positive area versus total lesion area in percentage. Representative images are shown to the right. Scale: 200 µm, inset scale: 100 µm. (**E**) Lesion CD4^+^ T-cell number per mm^2^. Representative images are shown to the right. Scale: 100 µm. (**F**) Lesion MHC class-II-positive area versus total lesion area in percentage. Representative images are shown to the right. Scale: 100 µm. Numbers and genotypes of mice are indicated in the legends.

We measured lesion contents of macrophages, CD4^+^ T cells, and MHC-II levels as indication of inflammatory cell infiltration and inflammation according to previously described methods^28^. Compared with the *Apoe* ^−/−^ *Tlr7* ^+/+^ control mice, aortic arches from *Apoe* ^−/−^ *Tlr7* ^−/−^ mice had significantly smaller Mac-3-positive macrophage areas (Fig. 3A), fewer lesion CD4^+^ T cells (Fig. 3B), and smaller MHC-II-positive areas (Fig. 3C). Significant smaller aortic arch atherosclerotic lesion sizes in *Apoe* ^−/−^ *Tlr7* ^−/−^ mice than those in *Apoe* ^−/−^ *Tlr7* ^+/+^ control mice may account for reduced lesion contents of macrophages, CD4^+^ T cells, and MHC-II-positive areas from *Apoe* ^−/−^ *Tlr7* ^−/−^ mice. When Mac-3-positive macrophage areas were expressed as percentage of total lesion areas, the macrophage contents in *Apoe* ^−/−^ *Tlr7* ^+/+^ control mice remained higher than those in *Apoe* ^−/−^ *Tlr7* ^−/−^ mice, but such a difference did not reach statistical significance (26.27 ± 11.34% *vs*. 10.50 ± 2.51%, *P* = 0.141) (Fig. 3D). Similarly, when CD4^+^ T-cell numbers were expressed as those of each mm^2^, we found comparable lesion CD4^+^ T-cell contents between *Apoe* ^−/−^ *Tlr7* ^+/+^ control mice and *Apoe* ^−/−^ *Tlr7* ^−/−^ mice (14.90 ± 3.45 number/mm^2^ *vs*. 18.10 ± 3.30 number/mm^2^, *P* = 0.341) (Fig. 3E). However, when MHC-II-positive areas were expressed as percentage of total lesion areas, we still saw significantly higher lesion MHC-II contents in *Apoe* ^−/−^ *Tlr7* ^+/+^ control mice than in *Apoe* ^−/−^ *Tlr7* ^−/−^ mice (42.13 ± 10.11% *vs*. 5.67 ± 1.03%, *P* = 0.002) (Fig. 3F).

**Figure 4:**
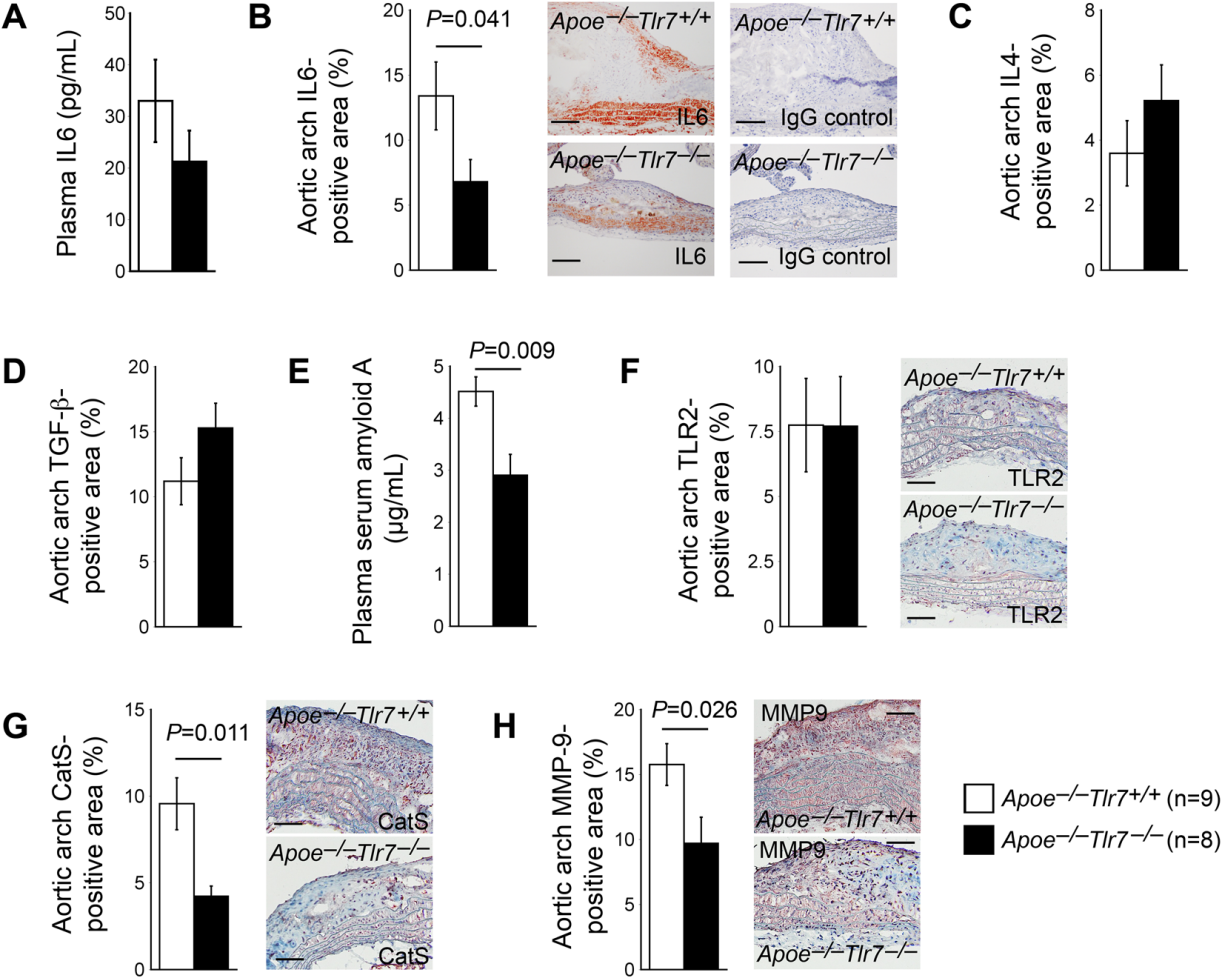
TLR7 deficiency changes aortic arch atherosclerotic lesion inflammatory cytokine and protease expression in male *Apoe* ^−/−^ mice. (**A**) Plasma IL6 level. (**B**) Lesion IL6-positive area percentage. Representative images are shown in the middle panels. Rabbit IgG isotype control staining results from parallel sections are show in the right panels. Scale: 200 µm. (**C**) Lesion IL4-positive area percentage. (**D**) Lesion TGF-β-positive area percentage. (**E**) Plasma SAA level. Lesion TLR2-positive area (**F**), CatS-positive area (**G**), and MMP-9-positive area (**H**) percentages. Representative images are shown to the right panels. Scales: 100 µm. Numbers and genotypes of mice are indicated in the legends.

IL6 is an important TLR7 downstream inflammatory product. TLR7 activation increases IL6 production^33, 34^. IFN-γ is another common pro-inflammatory cytokine in atherosclerosis. TLR7 activation can also indirectly produce IFN-γ^35, 36^. In contrast, TLR7 activation decreases TGF-β expression^37^. In turn, TGF-β inhibits TLR7 downstream pro-inflammatory molecule expression^38, 39^. IL4 is an anti-inflammatory cytokine that regulates TLR7 responses to viral infections^40^ or B-cell activation^41^. Therefore, TLR7 deficiency may attenuate atherosclerosis (Fig. 2A and B) by altering the production of these pro- and anti-inflammatory cytokines. Mouse plasma IFN-γ levels were undetectable by ELISA. Plasma IL6 levels from *Apoe* ^−/−^ *Tlr7* ^−/−^ mice were lower than those from the *Apoe* ^−/−^ *Tlr7* ^+/+^ littermates, but such a difference did not reach statistical significance (Fig. 4A). In the aortic arches, however, lesion IL6-positive areas were significantly lower in *Apoe* ^−/−^ *Tlr7* ^−/−^ mice than those in *Apoe* ^−/−^ *Tlr7* ^+/+^ mice (Fig. 4B, left and middle panels). Rabbit anti-mouse IL6 antibody specificity in mouse aortic arch immunostaining was verified using rabbit IgG isotype to stain the parallel sections and showed no immunoreactive signals (Fig. 4B, right panels). Lesion anti-inflammatory cytokines IL4- and TGF-β-positive areas were slightly elevated in aortic arches from the *Apoe* ^−/−^ *Tlr7* ^−/−^ mice, but such changes were also not statistically significant, compared with those from the *Apoe* ^−/−^ *Tlr7* ^+/+^ littermate control mice (Fig. 4C,D). Consistent with the hypothesis that TLR7 deficiency reduces lesion or systemic inflammation, plasma inflammation marker SAA was found significantly lower in *Apoe* ^−/−^ *Tlr7* ^−/−^ mice than in *Apoe* ^−/−^ *Tlr7* ^+/+^ control mice (Fig. 4E).

Both endolysosomal TLRs (e.g. TLR7, and TLR9) and cell surface TLRs (e.g. TLR2 and TLR4) share the same MyD88 and IRAK4/TRAF6 signaling pathways that lead to the production of pro-inflammatory cytokines^22, 42^. TLR7 deficiency is expected to reduce the production of lesion pro-inflammatory IL6 (Fig. 4B). Yet, it is also possible that endolysosomal TLR7 deficiency may affect the expression of cell surface TLRs. Therefore, reduced lesion IL6 (Fig. 4B) may come not only from TLR7 deficiency but also impaired expression of cell surface TLRs. We tested this possibility by immunostaining aortic arch atherosclerotic lesion TLR2 expression and did not detect significant differences in TLR2 expression in lesions between the *Apoe* ^−/−^ *Tlr7* ^−/−^ and *Apoe* ^−/−^ *Tlr7* ^+/+^ control mice (Fig. 4F). Instead, we found that atherosclerotic lesions from *Apoe* ^−/−^ *Tlr7* ^−/−^ mice contained significantly lower levels of both CatS (Fig. 4G) and MMP-9 (Fig. 4H), two common proteases that have been reported from atherosclerotic lesions, up-regulated by inflammatory cytokines, and play detrimental roles in atherogenesis^43–45^.

### TLR7 deficiency reduced lesion SMC loss and cell death

**Figure 5:**
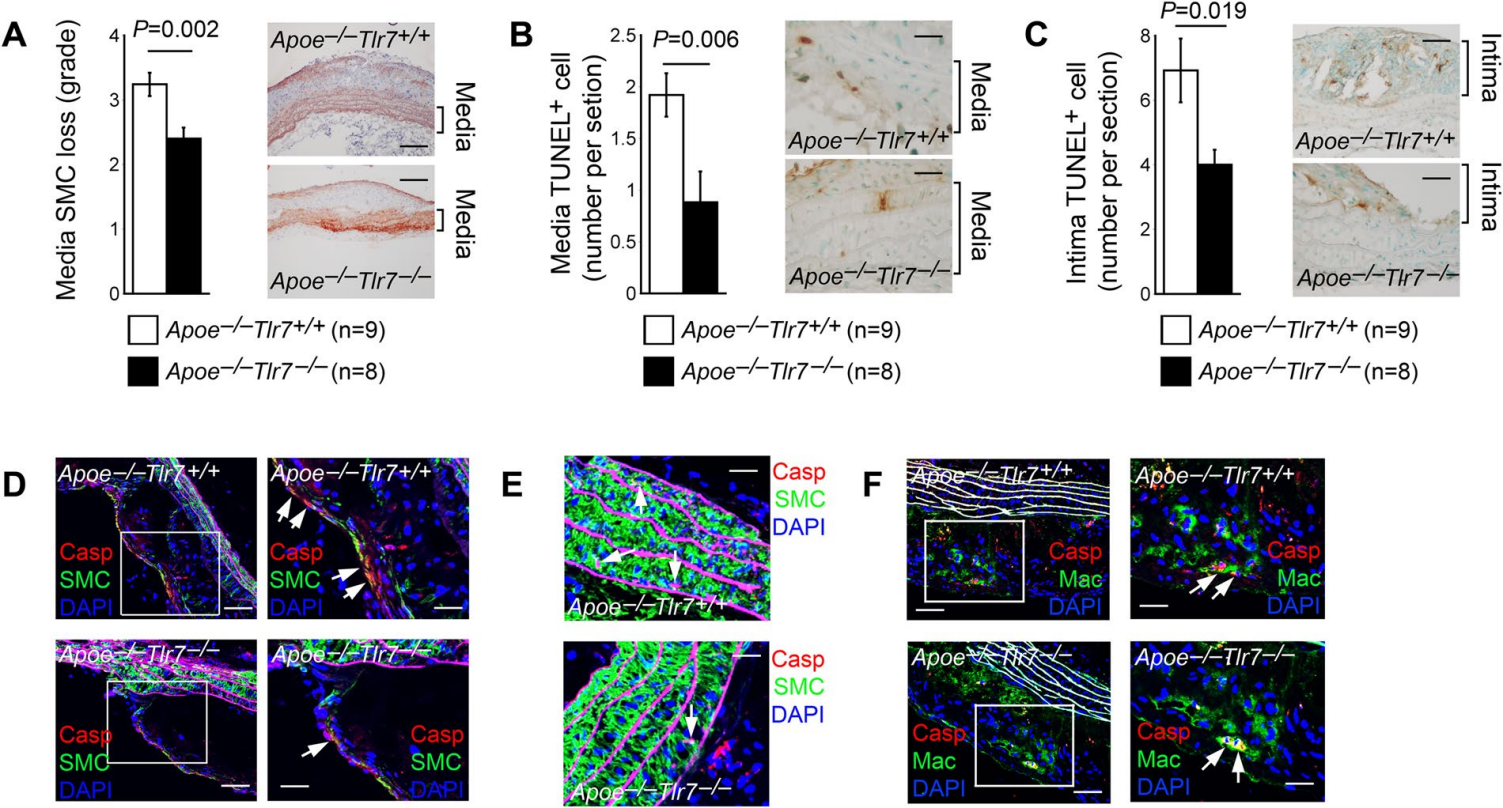
TLR7 deficiency reduces aortic arch atherosclerotic lesion SMC loss and cell apoptosis in male *Apoe* ^−/−^ mice. (**A)** Media SMC loss grade. Scale: 200 µm. (**B)** Media TUNEL-positive apoptotic cell number per section. Scale: 100 µm. (**C)** Intima TUNEL-positive apoptotic cell number per section. Scale: 100 µm. Media with clear elastic fibers and intima are framed. Representative images are shown to the middle panels. (**D)** Immunofluorescent double staining detected cleaved caspase-3 (Casp)-positive apoptotic SMC in the intima. Scale: 200 µm. Inset scale: 100 µm. (**E**) Cleaved caspase-3 (Casp)-positive apoptotic SMC in the media. Scale: 100 µm. (**F**) Cleaved caspase-3 (Casp)-positive apoptotic macrophages (Mac) in the lesions. Scale: 200 µm. Inset scale: 100 µm. Arrows indicate apoptotic cells. Numbers and genotypes of mice are indicated in the legends.

SMCs are the main cell type in the arterial wall and maintain the tissue phenotype and functional plasticity. These cells may switch their contractile phenotype into pro-inflammatory phenotype during atherogenesis-mediated inflammation, thereby undergoing apoptosis and cell loss^46^. SMC loss is associated with plaque rupture and lesion outward remodeling, and potential aneurysm formation^47, 48^. Indeed, plaques from patients with atherosclerosis with unstable symptoms showed higher levels of apoptosis than those with stable lesions^49^. In aortic arches from *Apoe* ^−/−^ *Tlr7* ^−/−^ mice, we found significantly lower grade of SMC loss than that from *Apoe* ^−/−^ *Tlr7* ^+/+^ control mice (Fig. 5A). As expected, we detected significantly fewer TUNEL-positive apoptotic cells in the aortic arch media (Fig. 5B) and intima (Fig. 5C) from *Apoe* ^−/−^ *Tlr7* ^−/−^ mice than those from *Apoe* ^−/−^ *Tlr7* ^+/+^ mice. To determine the cell types that underwent apoptosis in atherosclerotic lesions from these mice, we performed immunofluorescent double staining using anti-mouse cleaved caspase-3 and antibodies against SMC α-smooth muscle actin and macrophage Mac-2. Cleaved caspase-3 and α-actin double positive apoptotic SMCs were detected in both the intima (Fig. 5D) and media (Fig. 5E) from *Apoe* ^−/−^ *Tlr7* ^+/+^ and *Apoe* ^−/−^ *Tlr7* ^−/−^ mice. We also detected cleaved caspase-3 and Mac-2 double positive apoptotic macrophages in atherosclerotic lesions from both *Apoe* ^−/−^ *Tlr7* ^+/+^ and *Apoe* ^−/−^ *Tlr7* ^−/−^ mice (Fig. 5F).

### TLR7 deficiency did not affect arterial wall matrix protein expression or plasma lipoproteins

**Figure 6:**
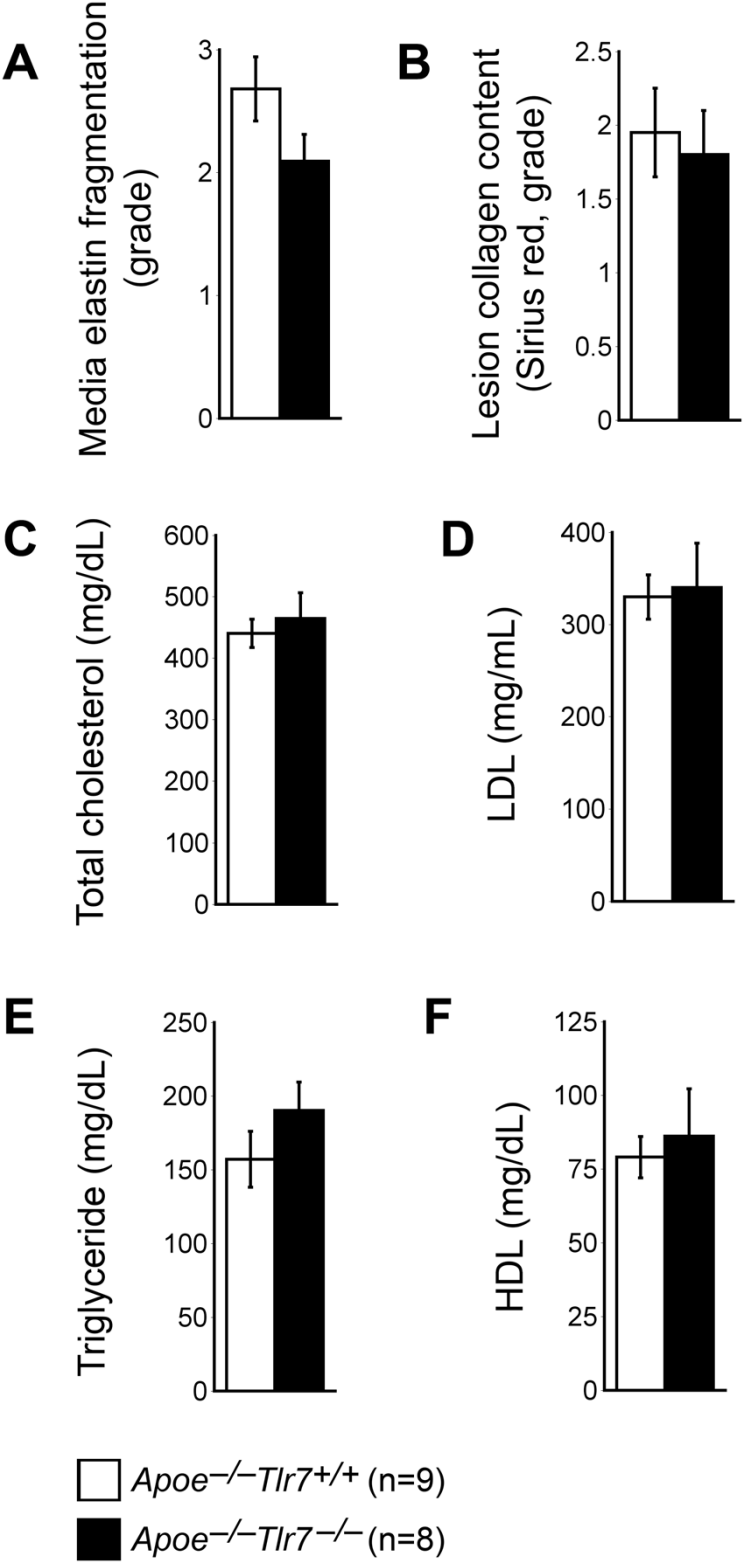
TLR7 deficiency does not affect aortic arch lesion matrix proteins or plasma lipid profile. (**A)** Lesion elastin fragmentation grade. (**B)** Lesion Sirius red-positive collagen grade. (**C–F)** Plasma total cholesterol, LDL, triglyceride, and HDL levels. Numbers and genotypes of mice are indicated in the legends.

Besides apoptosis, media SMC loss may also be associated with elastin degradation^50^. Collagen is also a major component of the fibrotic cap that maintains the cap strength and integrity^51^. While CatS is a potent elastase^52^, MMP-9 has both elastinolytic and collagenolytic activities^53–55^. Reduced expression of lesion CatS and MMP-9 in *Apoe* ^−/−^ *Tlr7* ^−/−^ mice may reduce lesion elastin fragmentation and increase collagen deposition. Yet, both elastin fragmentation and collagen contents in the aortic arches from both *Apoe* ^−/−^ *Tlr7* ^−/−^ and *Apoe* ^−/−^ *Tlr7* ^+/+^ mice did not differ (Fig. 6A,B), suggesting that the reduced media SMC loss in *Apoe* ^−/−^ *Tlr7* ^−/−^ mice was not due to changes in arterial wall elastin and collagen contents, although expression of both CatS and MMP-9 was reduced in lesions from these mice. Reduced lesion expression of these proteases in *Apoe* ^−/−^ *Tlr7* ^−/−^ mice may contribute to reduced lesion cell apoptosis as both are known to promote cell apoptosis^56, 57^. Elevated plasma total cholesterol, LDL, and triglyceride levels, and reduced plasma HDL are associated with the development of atherosclerosis in humans and mice^58, 59^. After consuming an atherogenic diet for 3 months, both *Apoe* ^−/−^ *Tlr7* ^−/−^ and *Apoe* ^−/−^ *Tlr7* ^+/+^ mice had comparable levels of plasma total cholesterol, LDL, triglyceride, and HDL (Fig. 6C–F).

## Discussion

TLR7 is an endolysosome membrane receptor that mediates endogenous antigen or viral/bacterial ssRNA-induced inflammation^14, 15, 60^. This study demonstrated a detrimental role of TLR7 in diet-induced atherosclerosis in *Apoe* ^*−/−*^ mice by increasing lesion inflammation (MHC-II), lesion pro-inflammatory cytokine expression (IL6), lesion protease expression (CatS, and MMP-9), and systemic inflammation (plasma SAA). However, our observations are controversial to what were reported in *Apoe* ^−/−^ mice that consumed a chow diet^21^, although this current study agrees with the observations that TLR7 antagonist blocked macrophage activation, foam cell formation, and neointima development in a femoral artery cuff placement-induced neointima thickening in ApoE*3-Leiden mice^23^. Our study also agrees with the conclusion from the hypercholesterolemic rabbits, in which TLR7 activation with imiquimod increased collar placement-induced carotid artery atherosclerotic lesion intima area, lipid deposition, proteoglycan level, macrophage contents, T cell contents, and angiogenesis^24^.

In male *Apoe* ^−/−^ mouse spontaneous atherosclerosis from the study by Salagianni *et al*.^21^, atherosclerotic lesion in the aortic root was enlarged in the absence of TLR7, along with elevated lesion lipid and macrophage contents, compared with the *Apoe* ^−/−^ control mice. In our study, after feeding the same male *Apoe* ^−/−^ *Tlr7* ^−/−^ mice an atherogenic diet, we detected reduced atherosclerosis and lipid deposition in both aortic arch and root from these mice, compared with the *Apoe* ^*−/−*^ *Tlr7* ^+/+^ littermates. It is possible that such discrepancies between the two studies were because of the differences in diet components. Dietary and commensal sources of PAMPs may activate TLR7 differently between chow and high cholesterol diets^61^. Indeed, similar atherosclerosis phenotype changes because of diet differences (chow diet *versus* atherogenic diet) have been reported previously in the same male IL18-deficient *Apoe* ^−/−^ (*Apoe* ^−/−^ *Il18* ^−/−^) mice. When mice were fed a chow diet and analyzed at 24 weeks of age, spontaneous atherosclerotic lesions were significantly reduced in *Apoe* ^−/−^ *Il18* ^−/−^ mice, compared with those in *Apoe* ^−/−^ control mice^62^. In contrast, when the same mice were fed a high cholesterol atherogenic diet at 5 weeks of age for 12 weeks, *Apoe* ^−/−^ *Il18* ^−/−^ mice developed significantly larger atherosclerotic lesions than did the *Apoe* ^−/−^ mice^63^. It was hypothesized that atherogenic diet may alter local and systemic inflammation^63^. The role of endolysosomal TLR in atherosclerosis has been controversial among all tested members. In atherogenic diet-fed *Ldlr* ^−/−^ and *Apoe* ^−/−^ mice, genetic depletion of TLR3 protected the mouse aortic wall from medial degradation with increased lesion collagen deposition and medial SMC content, although TLR3 deficiency did not affect lesion areas^7^. In contrast, when *Apoe* ^−/−^ mice were fed a chow diet for 15 weeks, TLR3 deficiency increased aortic lesion atherosclerotic lesion area^12^. A major difference between the two studies is the diet. However, diet alone may not explain the discrepancies between these studies. In *Apoe* ^−/−^ mice on a chow diet, TLR9 antagonist reduced aortic root atherosclerosis with decreased lesion inflammatory cell accumulation and increased lesion collagen and SMCs^64^. In ApoE*3 Leiden mice being fed a chow diet, TLR9 antagonist also reduced femoral artery cuff placement-induced neointima thickening and foam cell formation^23^. When *Apoe* ^−/−^ mice were on an atherogenic diet for 7 weeks, TLR9 activation with a high dose of its agonist CpG oligodeoxynucleotide (ODN)1826 enhanced atherosclerosis in the aortic root^65^. All these observations, regardless of diet types, point to a detrimental role of TLR9 in atherosclerosis. However, in an independent study of *Apoe* ^−/−^ mice on an atherogenic diet for 8 weeks, TLR9 deficiency (*Apoe* ^−/−^ *Tlr9* ^−/−^) increased aortic root atherosclerosis and lesion CD4^+^ T-cell content. CD4^+^ T-cell depletion in these mice reduced atherosclerosis, suggesting a role of TLR9 in regulating CD4^+^ T cells in atherosclerosis. In the same study, TLR9 activation with its agonist CpG ODN1668 decreased aortic root atherosclerosis^13^. These observations suggest a protective role of TLR9 in atherosclerosis. Obviously, the use of different diets may not explain all the discrepancies in the role of TLR9 in atherosclerosis and additional mechanisms may be involved and merit further investigation. A high cholesterol atherogenic diet is commonly used to produce atherosclerosis in atherosclerosis-prone *Apoe* ^−/−^ mice or in *Ldlr* ^−/−^mice^66–69^. Such diet may lead to steatosis and systemic inflammation, as part of its mechanism in atherosclerosis, abdominal aortic aneurysm, obesity, and diabetes studies^66–72^. Reduced atherosclerotic lesion IL6 expression and plasma SAA levels in *Apoe* ^−/−^ *Tlr7* ^−/−^ mice compared with those from *Apoe* ^−/−^ control mice from our study supported a role of TLR7 in regulating local and systemic inflammation. An atherogenic diet may affect TLR7 activation cascade, thereby changing downstream inflammatory molecule expression and atherogenesis. This hypothesis is consistent to several prior studies. For example, in C57BL/6 wild-type mice, TLR7 activation with R848 induced the production of IL10, IL6, TNF-α, and IL12 in a dose- and time-dependent manner^73^. In mouse macrophages, TLR7 activation with imiquimod induced macrophage production of IL6, MCP-1, RANTES (regulated on activation, normal T cell expressed and secreted), and TNF-α^24^. Effector T cells from imiquimod-treated squamous cell carcinomas produced more IFN-γ, granzyme, and perforin, and less IL-10 and TGF-β than T cells from untreated tumors^74^. All these data indicate a pro-inflammatory role of TLR7 in macrophages and lymphocytes. However, we cannot explain why macrophages from *Tlr7* ^−/−^ mice responded to TLR2 ligand lipoteichoic acid and produced significantly more MCP-1, IL6, and IL10 than those from wild-type control mice in a study from Salagianni *et al*.^21^.

Different from endolysosomal TLRs, cell surface TLRs such as TLR2 and TLR4 have been proven detrimental in atherosclerosis from most tested experimental models. In *Apoe* ^−/−^ mice on an atherogenic diet or a chow diet, deficiency of TLR4 or its signaling molecule MyD88 reduced aortic atherosclerosis, lesion lipid and macrophage contents, and peripheral IL1α, IL2 and MCP-1 levels^75, 76^. Similar observations were made in *Ldlr* ^−/−^ mice. Deficiency of TLR4 reduced atherosclerosis and plasma cholesterol and triglyceride, although did not affect obesity, hyperinsulinemia, or glucose intolerance after mice were fed a diabetogenic diet^8^. Similarly, TLR2-deficient *Apoe* ^−/−^ mice on a chow diet or an atherogenic diet or *Apoe* ^−/−^ mice on a chow diet but treated with an anti-mouse TLR2 antibody all demonstrated reduced atherosclerotic lesion size, lipid accumulation, macrophage and MCP-1 contents, and expression of cytokines (TNF-α and IL6) and associated transcription factors (NF-κB and STAT3)^77, 78^. It is possible that dietary and commensal sources of PAMPs from mice fed a chow and a high cholesterol diets may activate endolysosomal TLRs differently from cell surface TLRs^61^, a hypothesis that was not tested in this study.

The exact ligands that activate TLR7 during atherogenesis remain unknown. This is an interesting and important topic for further exploration. Endogenous TLR7 ligands include mainly ssRNA such as those from viral infections^79–81^, or even single nucleotide such as guanosine (G)/2′-deoxyguanosine (dG)^82^. Such nucleotide(s) in atherosclerotic lesions may activate TLR7 and promote atherosclerosis. For example, hepatitis C virus RNA and active cytomegalovirus were found in human atherosclerotic lesions^83, 84^. Hepatitis C infection facilitates human atherosclerosis^85^ and herpesvirus infection accelerates atherosclerosis in the *Apoe* ^−/−^ mice^86^. It is possible that these virus affects atherosclerosis by activating TLR7. Nevertheless, results from this study support a pro-inflammatory role of TLR7 in atherogenic diet-induced atherosclerosis in *Apoe* ^−/−^ mice. It is possible that TLR7 antagonists may have therapeutic potential in atherosclerosis and possibly other cardiovascular diseases^87^.
